# Multi-compartment profiling of donor-derived microRNAs during liver graft procurement: a prospective feasibility study

**DOI:** 10.3389/ti.2026.17090

**Published:** 2026-10-06

**Authors:** Mar Dalmau, Ramón Charco, Itxarone Bilbao, Cristina Dopazo, Mireia Caralt, J. A. Molino, Alberto Sandiumenge, Gonzalo Sapisochin, Concepción Gómez-Gavara

**Affiliations:** 1 Universitat Autonoma de Barcelona, Barcelona, Spain; 2 Hepatobiliopancreatic Surgery and Liver Transplantation Unit, Hospital Universitari Vall d’Hebron, Barcelona, Spain; 3 Quirúrgica Cirujanos Asociados, Centro Medico Teknon, Barcelona, Spain; 4 CIBEREHD, Centro de Investigacion Biomedica en Red Enfermedades Hepaticas y Digestivas, Madrid, Spain; 5 Hepatobiliopancreatic Surgery and Liver–Pancreas Transplantation Unit, Hospital Clinic de Barcelona, Barcelona, Spain

**Keywords:** liver transplantation, microRNAs, organ procurement, normothermic regional perfusion, graft injury

## Abstract

Liver grafts from donors after brain death (DBD) and donors after circulatory death (DCD) are exposed to distinct mechanisms of injury. We evaluated the feasibility of multi-compartment profiling of liver injury-associated microRNAs (miRNAs) across serum, liver tissue, and preservation fluid in deceased donor liver grafts and explored compartment-specific expression patterns according to donor type. In this prospective single-centre exploratory study, miR-122, miR-148a, miR-22, miR-222, and miR-155 were quantified by RT-qPCR in serum, liver tissue, and preservation fluid from 10 DBD and 10 DCD donors, including paired serum sampling before and during abdominal normothermic regional perfusion (aNRP). Three declined steatotic DBD grafts were analysed descriptively. A total of 79 samples were analysed. Baseline serum miRNA profiles and post-transplant outcomes were comparable between DBD and DCD grafts. Distinct compartment-specific expression patterns were observed, particularly in preservation fluid, where DCD grafts recovered following aNRP showed lower expression of miR-148a, miR-222, and miR-22 than DBD grafts. In DCD donors, serum during aNRP showed lower miR-148a expression. Exploratory descriptive analyses of declined steatotic grafts identified miRNA patterns consistent with hepatocellular injury. These findings demonstrate the feasibility of multi-compartment donor-derived miRNA profiling and provide a framework for future studies evaluating compartment-specific molecular markers for graft assessment.

## Graphical Abstract

**Figure:**
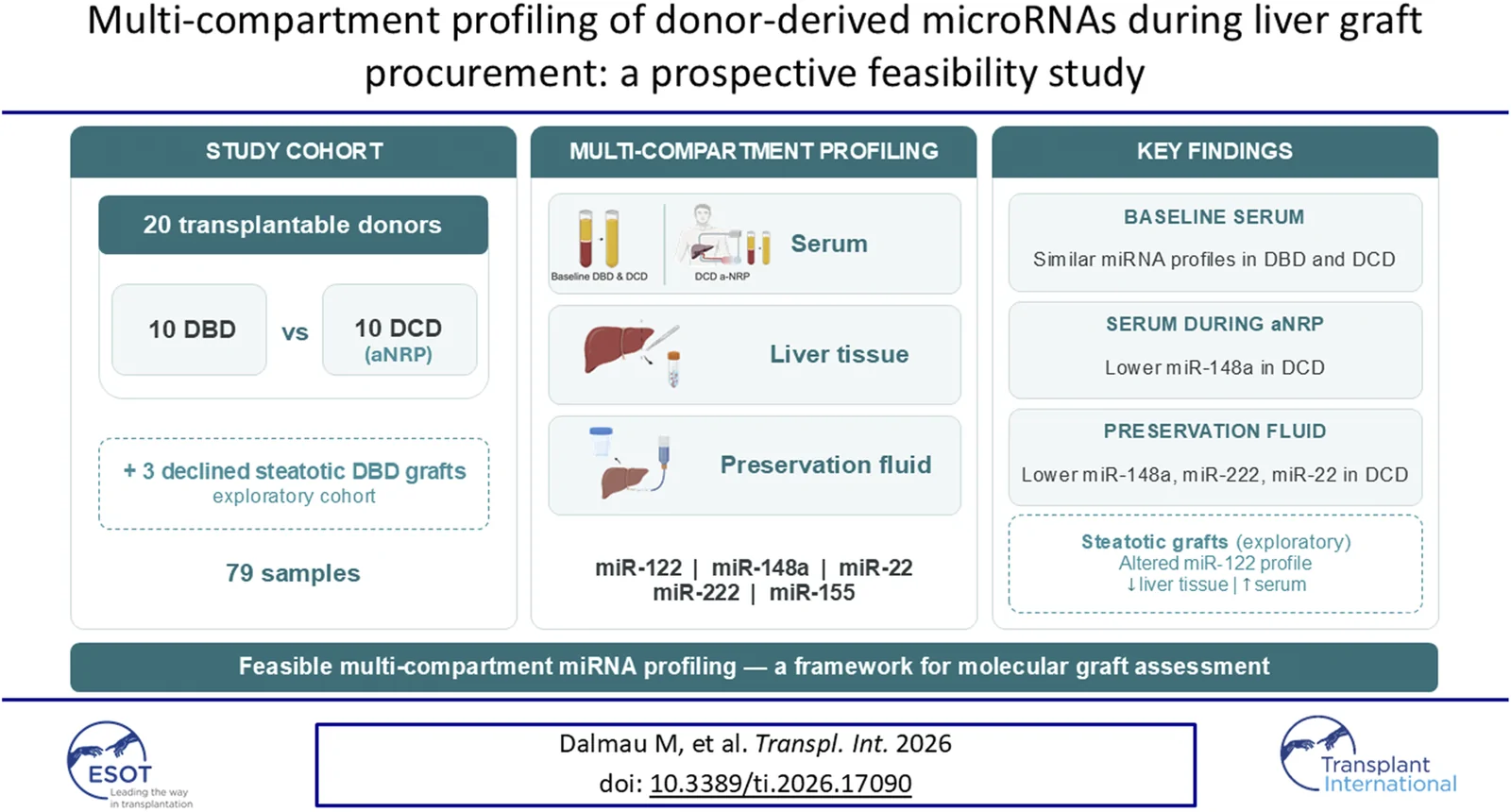

## Introduction

The main source of organs for transplantation continues to be deceased donors after brain death (DBD). However, due to the persistent shortage of organs for liver transplantation, donation after circulatory death (DCD) has expanded substantially over the past decade and currently accounts for more than 25% of deceased organ donation worldwide and over 50% of liver transplants performed in countries where this donation pathway is established [1].

In its early implementation, liver transplantation from DCD donors was associated with inferior outcomes, largely driven by a higher incidence of biliary complications [2]. More recently, the introduction of *in situ* abdominal normothermic regional perfusion (aNRP) and *ex situ* machine perfusion techniques have significantly mitigated ischemic injury and hepatobiliary hypoxia during warm ischemia, resulting in transplant outcomes comparable to those achieved with DBD grafts [3, 4].

Although DBD donors, in contrast to DCD donors, are not exposed to warm ischemia, catastrophic brain injury leading to brain death triggers profound systemic pathophysiological changes. These include hemodynamic instability with hypotension and hypoperfusion, which may adversely affect distant organs [5]. In addition, brain death induces a marked pro-inflammatory state characterized by cytokine release and leukocyte activation, contributing to increased graft immunogenicity and higher rates of acute rejection when compared with living donation and DCD transplantation [6, 7]. The relative contribution of these mechanisms to liver graft quality and post-transplant outcomes in DBD versus DCD donors remains incompletely understood.

MicroRNAs (miRNAs) have emerged as stable, tissue-enriched biomarkers that can be detected both intracellularly and in circulation, where they are protected from degradation through association with protein complexes, lipoproteins, and, to a lesser extent, extracellular vesicles. In the context of liver transplantation, altered miRNA expression has been associated with hepatobiliary injury, ischemia–reperfusion damage, and adverse postoperative outcomes [8–12]. However, data on liver-derived miRNA expression in donor samples and preservation fluids remain limited, despite their potential to provide objective, molecular-level information on graft condition prior to transplantation.

The primary aim of this study was to evaluate the feasibility of multi-compartment profiling of liver injury–associated miRNAs in deceased donor liver grafts and to explore compartment-specific expression patterns according to donor type.

## Patients and methods

### Study population

Between October 2019 and May 2021, liver tissue biopsies, preservation fluid, and serum samples were prospectively collected from adult deceased organ donors at a tertiary referral transplant centre. During the study period, samples were obtained from 38 deceased donors. Three grafts were declined for transplantation based on intraoperative assessment of significant steatosis; moderate-to-severe steatosis was subsequently confirmed histologically, and these grafts were analysed separately as an exploratory cohort. Of the remaining 35 transplantable donors, seven were excluded because one or more samples did not fulfill predefined RNA quality-control criteria for inclusion in the multi-compartment analysis, and eight donors were included in a parallel methodological study evaluating the effect of heparinase treatment on RT-qPCR analysis of heparinized donor samples [13]. The final analytical cohort therefore comprised 20 transplantable donors (10 DBD and 10 DCD) and three declined steatotic DBD grafts, representing a total of 79 biological samples across all compartments.

Transplantable liver grafts obtained from donors after brain death (DBD) and controlled donors after circulatory death (DCD) were analysed to characterize liver injury–associated microRNA (miRNA) expression profiles. This study was designed as a prospective exploratory study. Donors for retransplantation, paediatric donors (<18 years), and split-liver grafts were excluded.

Because moderate-to-severe hepatic steatosis is associated with increased susceptibility to ischemia–reperfusion injury and inferior early graft outcomes [14], an exploratory analysis was performed in the three declined steatotic DBD grafts to assess whether miRNA expression patterns differed from those of transplantable grafts. These grafts were analysed descriptively and were not included in recipient outcome analyses. Although these grafts were declined due to macroscopically significant steatosis, they were recovered and perfused following the same protocol as transplantable grafts, and biopsy-proven steatosis was subsequently confirmed histologically.

### Steatosis evaluation

The degree of hepatic steatosis was assessed histologically in liver biopsy specimens obtained after abdominal inspection in DBD donors and after initiation of abdominal normothermic regional perfusion (aNRP) in DCD donors. Steatosis was graded according to the percentage of hepatocytes containing fat droplets as absent to mild (<30%) or moderate to severe (≥30%), in accordance with established criteria [15].

### Sample collection and processing

The sample collection timeline is summarized in Figure 1. In DBD donors, a 10-mL blood sample was collected before laparotomy and before systemic heparin administration (300 IU/kg) and aortic cannulation (DBD_T0). In DCD donors, paired blood samples were obtained: the first prior to withdrawal of life-sustaining therapy (DCD_T0), followed by systemic heparinization at the onset of donor hypotension (systolic blood pressure <60 mmHg). After 5 min of asystole, abdominal normothermic regional perfusion (aNRP) was initiated via post-mortem cannulation, and a second blood sample was collected from the extracorporeal circuit (DCD_T1). The aNRP circuit was maintained for 90–120 min to allow assessment of graft viability.

**FIGURE 1:**
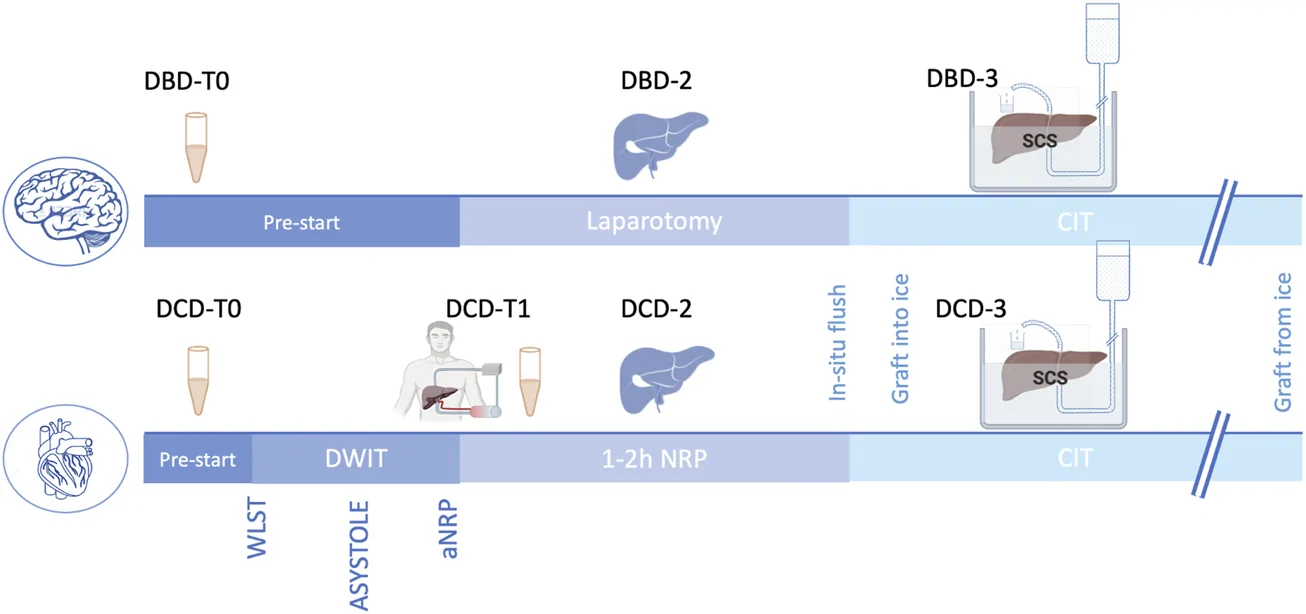
Sample collection timeline in donors after brain death (DBD) and controlled donors after circulatory death (DCD). In DCD donors, paired serum samples were obtained before withdrawal of life-sustaining therapy (T0) and during abdominal normothermic regional perfusion (aNRP, T1). Abbreviations: aNRP, abdominal normothermic regional perfusion; CIT, cold ischemia time; SCS, static cold storage; WLST, withdrawal of life-sustaining therapy.

This sampling strategy enabled partially paired, multi-compartment analysis within the same donor. The number of biological samples available for each analysis differed according to donor group and compartment, and the corresponding sample size is reported in each figure legend and supplementary table.

Liver wedge biopsies were obtained after abdominal inspection in both transplantable and discarded DBD donors and after initiation of aNRP in DCD donors. During organ procurement, abdominal organs were flushed with 8 L of IGL-1 preservation solution. At the back table, 30 mL of preservation fluid was collected from the hepatic veins following portal vein flushing with 100 mL of IGL-1.

Blood samples were collected into serum-separation tubes and centrifuged within 4 h of collection to isolate serum. Liver tissue, serum, and preservation-fluid samples were frozen at −80 °C at the end of the procurement procedure and stored until RNA extraction.

Sample processing and RNA isolation were adapted to each biological compartment, whereas downstream RT-qPCR and data analysis followed a standardized workflow, as previously described [13]. Heparinase I treatment was not applied, as no evidence of heparin-related RT-qPCR inhibition was observed in this cohort, in contrast to prior reports in other transplant settings [16].

Individual miRNA measurements that failed predefined assay quality-control criteria or were not consistently detectable were excluded from the corresponding analyses. Consequently, the effective sample size varies slightly according to the individual miRNA analysed.

### miRNA selection

Five miRNAs associated with liver injury and post-transplant complications (miR-122, miR-148a, miR-155, miR-22, miR-222) and the miR-122/miR-222 ratio were selected based on prior literature [10–12, 17–19]. Five endogenous miRNAs (miR-103a, miR-191, miR-30c, miR-16, and let-7a) were evaluated as candidate reference genes according to reported stability in liver disease–related studies [11, 20, 21] (Table 1).

**TABLE 1:** Endogenous miRNA PCR primer sets.

Primer sets from miRNA PCR assay Qiagen (Germany)	
miRNA target genes	hsa-miR-122-5p ref.#YP00205664 hsa-miR-148a-3p ref.#YP00205867 hsa-miR-155-5p ref.#YP00204308 hsa-miR-22-3p ref.#YP00204606 hsa-miR-222-3p ref.#YP00204551
miRNA reference genes	hsa-miR-103a-3p ref.#YP00204063 hsa-miR-191-5p ref.#YP00204306 hsa-miR-30c-5p ref.#YP00204783 hsa-miR-16-5p ref.#YP00205702 hsa-let-7a-5p ref.#YP00205727

### Post-transplant clinical outcomes

Clinical outcomes were analysed as secondary exploratory endpoints. Associations between miRNA levels and early allograft dysfunction within the first postoperative week were assessed [22], along with correlations with peak AST/ALT levels within the first 24 h after transplantation.

Early liver injury was defined as peak alanine or aspartate aminotransferase levels >1000 IU/L within the first 24 h after transplantation [23]. Additional outcomes included biliary complications (leak or stricture), biopsy-proven acute cellular rejection, and the Comprehensive Complication Index (CCI) within 90 days after transplantation.

The study was not powered to detect differences in clinical outcomes, and these analyses were considered exploratory.

### Statistical analysis

Descriptive statistics were used to summarize demographic and clinical variables. Categorical variables were compared using the chi-square or Fisher’s exact test. Continuous variables were analysed using Student’s t*-*test for normally distributed data or the Mann–Whitney *U* test for non-normally distributed data.

RT-qPCR data quality control excluded Ct values < 11 or >39. Mean Ct values were calculated from technical triplicates, and replicates with a standard deviation > 0.3 were refined by removing the outlier furthest from the mean, retaining at least two values per miRNA per sample. Samples with fewer than two valid replicates were excluded.

Ct values were normalized using the mean Ct of five endogenous reference miRNAs to account for differences in RNA input and reverse transcription efficiency. Normalized miRNA expression levels were represented as 2^-ΔCt values, where ΔCt was defined as the Ct of the target miRNA minus the mean Ct of the selected reference genes.

Relative differences between groups (fold changes) were calculated using the 2^-ΔΔCt method as described by Livak and Schmittgen [24]. Statistical comparisons were performed using normalized ΔCt values. Group comparisons were analysed using Student’s t-test or the Mann–Whitney U test, as appropriate. Paired serum samples from DCD donors (T0 versus T1) were analysed using a paired Student’s t-test. Associations between miRNA expression and peak transaminase levels were assessed using Spearman’s rank correlation. Because the declined steatotic graft subgroups comprised only three donors, these data were analysed descriptively, and no formal statistical inference was performed for this subgroup.

For visualization purposes, relative expression is presented as 2^-ΔCt in Figures 2, 3, whereas Figure 4 and Supplementary Figure S1 display normalized -ΔCt values corresponding to those used in the statistical analyses.

**FIGURE 2:**
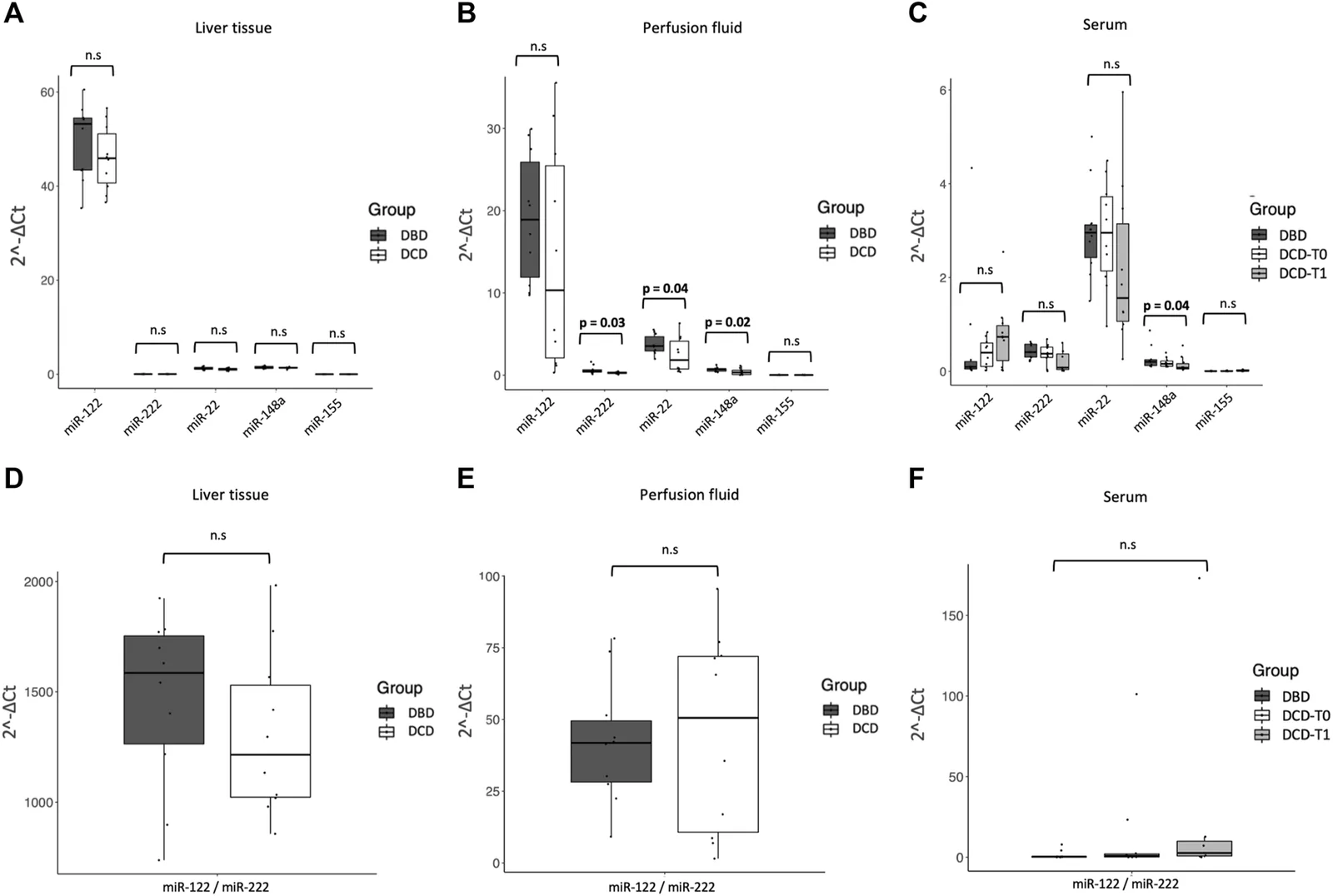
Relative miRNA expression in transplantable liver grafts. Relative miRNA expression (2^−ΔCt^) in **(A)** liver tissue (DBD, n = 10; DCD, n = 10), **(B)** preservation fluid (DBD, n = 10; DCD, n = 10), and **(C)** serum from DBD donors (DBD, n = 10) and paired serum samples from DCD donors collected before withdrawal of life-sustaining therapy (DCD-T0, n = 10) and during aNRP (DCD-T1, n = 10). Panels **(D–F)** show the miR-122/miR-222 ratio of the corresponding compartments. Boxplots represent the median and interquartile range, with whiskers indicating minimum and maximum values. Statistical significance is indicated by the corresponding *p-values.* The number of evaluable RT-qPCR measurements for each miRNA is provided in Supplementary Tables S1–S3.

**FIGURE 3:**
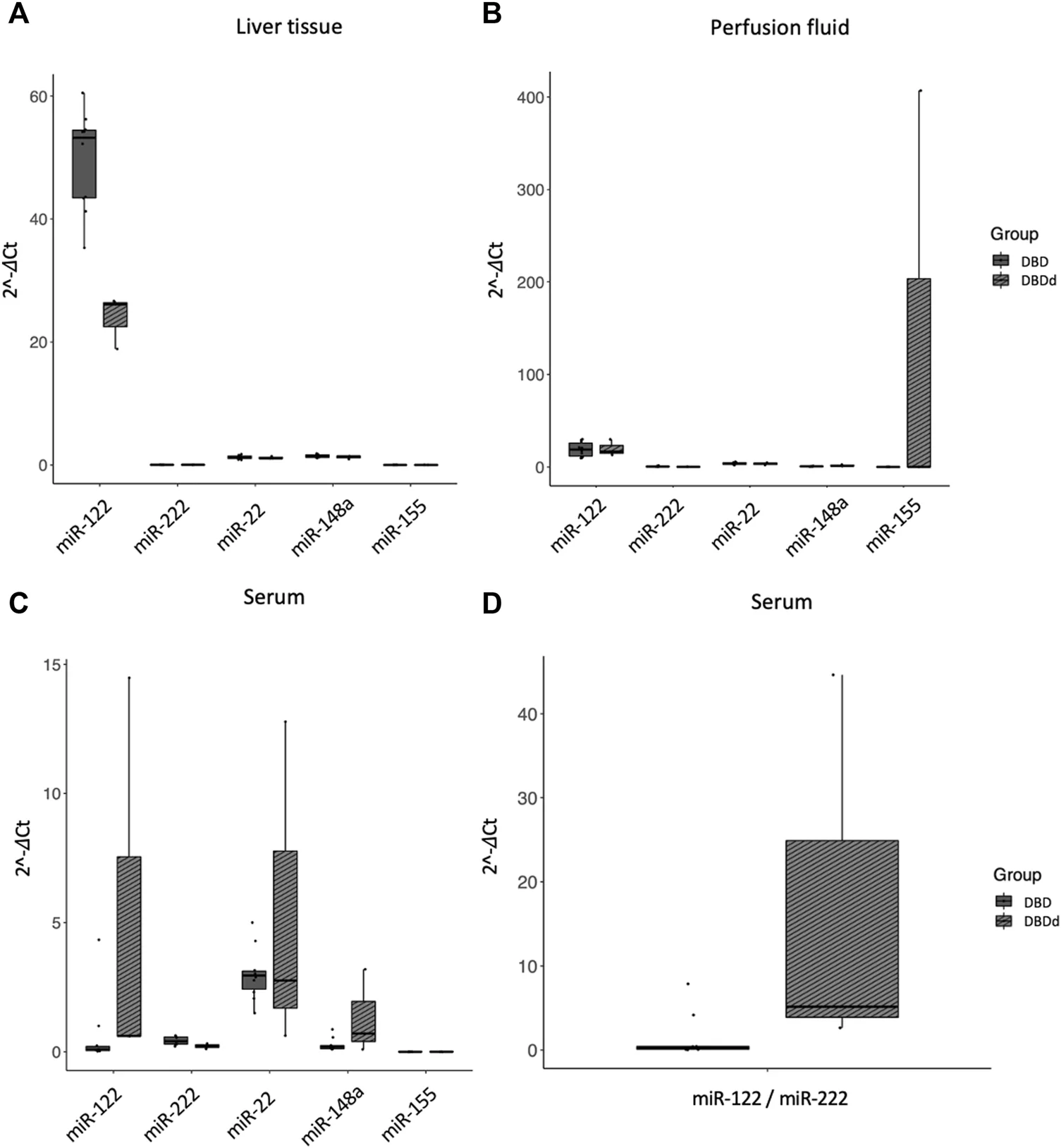
Relative miRNA expression in declined steatotic liver grafts (exploratory analysis). Relative miRNA expression (2−^ΔCt^) in **(A)** liver tissue (DBD, n = 10; declined DBD [DBDd], n = 3), **(B)** preservation fluid (DBD, n = 10; DBDd, n = 3), and **(C)** serum (DBD, n = 10; DBDd, n = 3). Panel **(D)** shows the serum miR-122/miR-222 ratio in the same donor groups. Because the declined steatotic cohort comprised only three grafts, this comparison is descriptive and no formal statistical inference was performed. Boxplots represent the median and interquartile range, with whiskers indicating minimum and maximum values.

**FIGURE 4:**
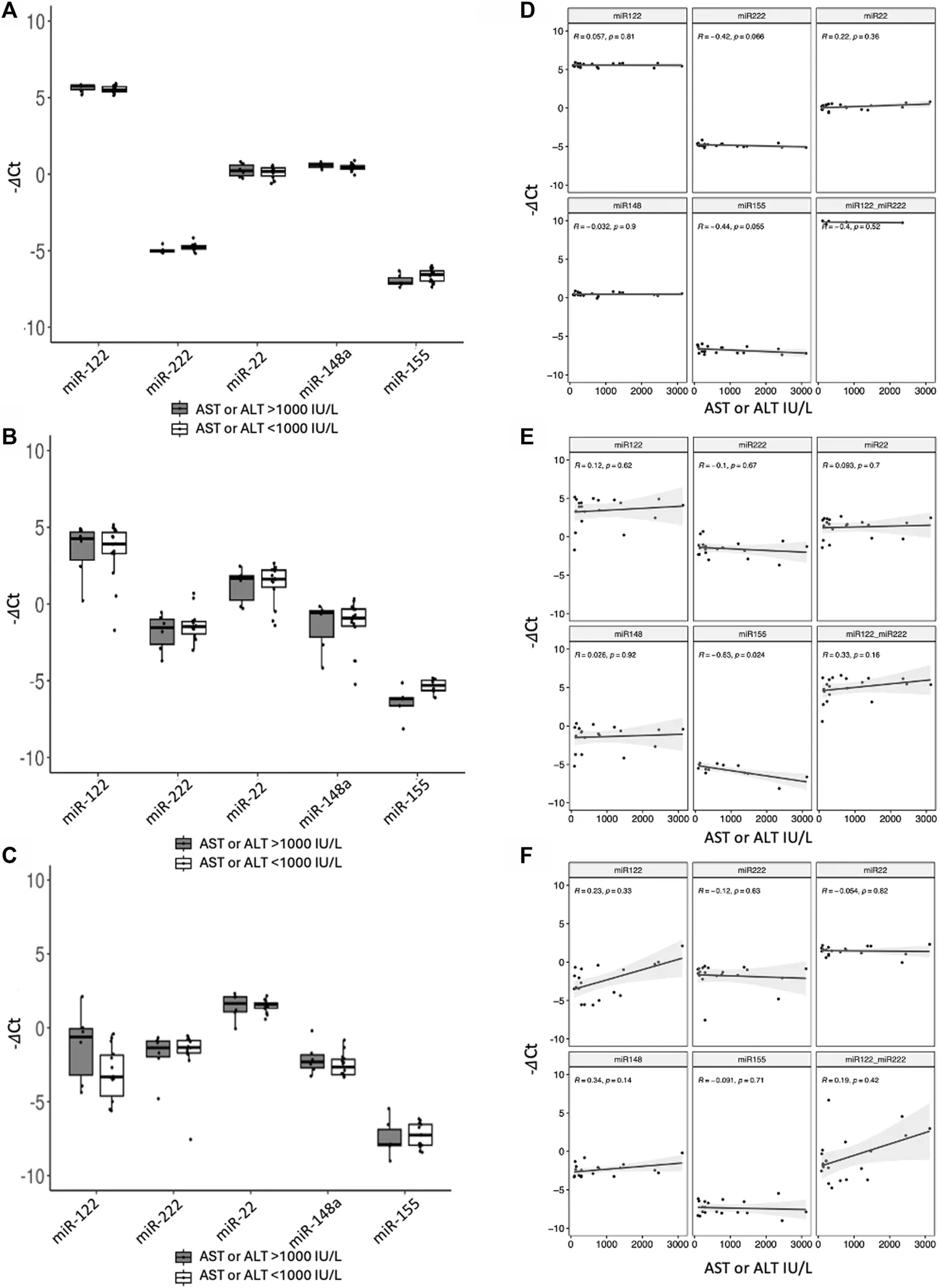
Association between donor miRNA expression and early post-transplant liver injury. Normalized miRNA expression represented as −ΔCt values in liver tissue **(A)**, preservation fluid **(B)**, and serum **(C)** from 20 transplantable grafts (10 DBD and 10 DCD), grouped according to peak AST and/or ALT levels >1000 IU/L within the first 24 h after transplantation. Panels **(D–F)** show Spearman correlations (r) between donor-derived miRNA expression and peak post-transplant transaminase levels. The number of evaluable RT-qPCR measurements for each miRNA is provided in Supplementary Tables S1–S3.

A p value < 0.05 was considered statistically significant. Given the exploratory nature of the study, the absence of adjustment for multiple testing, and the limited sample size, statistical analyses were intended to identify potential biological patterns rather than to provide confirmatory evidence. Statistical analyses were performed using R software (version 4.2.0).

### Normalization strategy assessment

Reference gene stability was evaluated using geNorm and NormFinder algorithms. geNorm ranked candidate genes according to stability (M values ≤ 1.5 indicating stable expression) and calculated pairwise variation (V_n_/_n+1_) to determine the optimal number of reference genes [25]. NormFinder assessed stability based on intra- and intergroup variation [26]. Four normalization strategies were tested (Table 2), and performance was evaluated by calculating the coefficient of variation of 2^−ΔCt values across all sample types. The synthetic spike-in cel-miR-39, absent in mammals, was included as an external control for RT-qPCR efficiency.

**TABLE 2:** Normalization strategies and corresponding reference miRNAs.

Normalization methods	Reference miRNA genes
Normalization with 1 endogenous gene (norm1end)	miR-191
Normalization with 2 endogenous genes (norm2end)	miR-191, miR-103
Normalization with 5 endogenous genes (norm5end)	miR-191, miR-103, miR-30c, miR-16, miR-let7a
Normalization with Cel-miR-39 (normir39)	Cel-miR-39

### Ethics

Ethical approval for this study was provided by the local institutional ethics committee (PR[AG]46/2019). All research was conducted in accordance with the ethical principles of the Declaration of Helsinki and the Declaration of Istanbul. Written informed consent was obtained from transplant recipients and donor relatives prior to inclusion. The study was registered at ClinicalTrials.gov (NCT06611046). Samples and clinical data were provided by an institutional Biobank (PT20/00107) and processed according to standard operating procedures.

## Results

### Donor and recipient characteristics

The final analytical cohort comprised 20 transplantable donors (10 DBD and 10 DCD) and three declined steatotic DBD grafts, representing a total of 79 biological samples (23 liver tissue, 23 preservation fluid, and 33 serum samples).

Donor and recipient characteristics are summarized in Table 3. No statistically significant differences were observed between DBD and DCD donors with transplantable grafts, or between recipient demographics, except for higher serum sodium levels in DBD donors. All transplantable grafts showed absent or mild steatosis on histological assessment. Post-transplant clinical outcomes, including early allograft dysfunction, biliary complications, acute rejection, and length of hospital stay, were comparable between groups.

**TABLE 3:** Donor and recipient characteristics and clinical outcome.

Donor variables	DBD (n = 10)	DCD (n = 10)	P value
Age (years)	65.5 (±11.7)	62.6 (±7.4)	0.24
Gender male/female (%)	4 (40)/6 (60)	4 (40)/6 (60)	1
BMI (kg/m^2^)	25.4 (±3.0)	27.8 (±4.7)	0.27
ICU length of stay (days)	3.70 (±3.1)	7.10 (±6.6)	0.13
Donor maintenance† (hours)	9.21 (±3.3)	NA	NA
Sodium (mEq/L)	147.7 (±7.2)	141.4 (±4.1)	**0.02**
AST (IU/L)	33.9 (±36.8)	37.1 (±33.9)	0.84
ALT (IU/L)	19.4 (±10.9)	25.7 (±14.4)	0.28
Bilirubin ≥1.2 mg/dL	2 (20)	0 (0)	0.21
WIT (min)	NA	27.00 (±8.9)	NA
f-WIT (min)	NA	22.60 (±6.5)	NA
Recipient variables			
Age (years)	59.70 (±8.3)	55.10 (±11.4)	0.32
Gender male/female (%)	6 (60)/4 (40)	8 (80)/2 (20)	0.63
BMI (kg/m^2^)	25.30 (±3.2)	26.81 (±4.57)	0.43
MELD	14.10 (±5.9)	18.00 (±10.0)	0.30
Total ischemic time (min)	349.5 (±54.9)	339.33 (±56.9)	0.70
Peak AST IU/L (7days)	1,016 (±996)	639 (±738)	0.35
Bilirubin mg/dL (7th POD)	4.02 (±4.24)	2.90 (±3.32)	0.52
INR (7th POD)	1.20 (±0.44)	1.00 (±0.04)	0.18
EAD	2 (20)	2 (20)	1
Length of stay (days)	12 (±5)	19 (±18)	0.34
Biliary complications	1 (10)	1 (10)	1
Acute rejection	4 (40)	4 (40)	1
CCI	10.59 (±5.97)	23.86 (±23.5)	0.11

Data are presented as n (%) or mean ± standard deviation (SD). †Donor maintenance refers to the time from brain death diagnosis to organ procurement. Abbreviations: BMI, body mass index; ICU, intensive care unit; WIT, warm ischaemic time; f-WIT, functional warm ischaemic time; AST, aspartate aminotransferase; ALT, alanine aminotransferase; INR, international normalised ratio; EAD, early allograft dysfunction; CCI, Comprehensive Complication Index. Bold values indicate statistically significant differences (*p* < 0.05).

### miRNA expression in transplantable liver grafts

#### Liver tissue

Among all miRNAs analysed, miR-122 showed the highest expression in liver tissue, with levels approximately 48-fold higher than reference genes. Expression levels were similar between donor types (DBD vs. DCD: 1.19-fold difference; p = 0.14; Figure 2A; Supplementary Table S1).

#### Preservation fluid

In preservation fluid, miR-122 was also the most abundantly expressed miRNA, with levels approximately 17-fold higher than reference genes. Expression tended to be higher in DBD compared with DCD grafts (2.76-fold difference; p = 0.08).

In contrast, miR-222, miR-22, and miR-148a showed lower expression in DCD preservation fluid compared with DBD grafts (fold changes 0.36, 0.45, and 0.33, respectively; p values 0.02–0.04), indicating a consistent pattern across these miRNAs (Figure 2B; Supplementary Table S2).

#### Serum

In DCD donors, serum obtained from the aNRP circuit after warm ischemia showed a reduction in miR-148a expression (0.45-fold; p = 0.04) compared with DBD serum. A non-significant increase in miR-122 expression was also observed (2.7-fold; p = 0.19). Pre-procurement serum miRNA levels did not differ between DBD and DCD donors. (Figure 2C; Supplementary Table S3).

The miR-122/miR-222 ratio was higher in DCD samples compared with DBD samples both before and after warm ischemia (2.8-fold and 7.8-fold, respectively), although these differences did not reach statistical significance (Figures 2D–F).

### Exploratory descriptive analysis of declined steatotic grafts

#### Donor characteristics

Three DBD liver grafts were declined for transplantation based on intraoperative subjective assessment of significant steatosis, which was subsequently confirmed as moderate-to-severe macrovesicular steatosis on histology (30%, 45%, and 60%). Donor characteristics are summarized in Table 4. Declined grafts had higher donor BMI compared with transplantable DBD grafts.

**TABLE 4:** Donor characteristics of transplantable and declined DBD donors with moderate-to-severe steatosis. Values are presented descriptively. Because the declined steatotic cohort comprised only three grafts, no formal statistical comparisons were performed.

Donor variables	DBD (n = 10)	DBDd (n = 3)
Age (years)	65.5 (±11.7)	55 (±3.6)
Gender male/female (%)	4 (40)/6 (60)	2 (66.6)/1 (33.3)
BMI (kg/m^2^)	25.4 (±3.0)	30.5 (±3.2)
ICU length of stay (days)	3.7 (±3.1)	1.7 (±1.2)
Donor maintenance (h)	9.2 (±3.3)	10.9 (±5.1)
Na (mEq/L)	147.7 (±7.2)	143.5 (±5.6)
AST (IU/L)	33.9 (±36.8)	118.0 (±152.4)
ALT (IU/L)	19.4 (±10.9)	136.7 (±82.5)
GGT (IU/L)	52.0 (±56.3)	21.0 (±16.2)
Bilirubin (mg/dL)	0.97 (±0.58)	0.53 (±0.35)

Data are presented as n (%) or mean ± standard deviation (SD). Abbreviations: BMI, body mass index; ICU, intensive care unit; AST, aspartate aminotransferase; ALT, alanine aminotransferase; GGT, gamma-glutamyl transferase.

#### miRNA expression

Exploratory analysis of the three declined steatotic DBD grafts revealed distinct miRNA expression patterns compared with transplantable DBD grafts (Figure 3; Table 4). In liver tissue, declined steatotic grafts showed lower miR-122 expression (0.48-fold relative to transplantable DBD grafts). In preservation fluid, miR-222 expression was lower (0.36-fold), resulting in a threefold higher miR-122/miR-222 ratio.

Serum miRNA expression was broadly comparable between groups, although declined grafts showed higher circulating miR-122 levels (12.9-fold) and an approximately 25-fold serum miR-122/miR-222 ratio. Given the limited numbers of declined grafts (n = 3), these findings are presented descriptively and should be considered hypothesis-generating.

### Donor miRNAs and early post-transplant liver injury

Among the 20 transplanted recipients, six developed peak AST and/or ALT levels >1000 IU/L within the first postoperative day, and four developed early allograft dysfunction.

miR-155 expression in preservation fluid showed an inverse correlation with peak transaminase levels (r = −0.63; p = 0.024), with a similar trend observed in liver tissue (r = −0.44, p = 0.055; Figure 4).

No significant associations were identified for other miRNAs.

In serum, grafts associated with higher transaminase levels (>1000 IU/L) showed higher miR-122 expression (3.7-fold; p = 0.13) and miR-122/miR-222 ratios (4.0-fold; p = 0.21), although these differences did not reach statistical significance. Similarly, donors of grafts that developed early allograft dysfunction showed higher serum miR-122 (6.4-fold; p = 0.09) and miR-122/miR-222 ratio (9.1-fold, p = 0.12; Supplementary Figure S1).

### Normalization strategy

All candidate reference miRNAs demonstrated acceptable stability, with geNorm M values below 1.5. In liver tissue, all pairwise variation values were below the recommended threshold, whereas greater variability was observed in serum and preservation fluid.

Normalization using the mean expression of five endogenous reference miRNAs provided the lowest variability across all sample types and was therefore used throughout the study (Supplementary Figures S2, S3).

## Discussion

The principal finding of this prospective study is that multi-compartment profiling of donor-derived miRNAs is feasible during contemporary liver graft procurement and provides compartment-specific expression patterns that vary by donor type. The main observations were that (i) transplantable DBD and DCD grafts showed comparable miRNA profiles in serum prior to procurement, (ii) distinct differences emerged in preservation fluid according to donor type, and (iii) exploratory analyses of three declined steatotic grafts revealed miRNA expression patterns consistent with hepatocellular stress.

A key strength of this study lies in its prospective multi-compartment sampling strategy, which allowed repeated measurements within the same donor across different biological matrices and reduced interindividual variability. In particular, the inclusion of aNRP circuit paired serum sampling in DCD donors before and during aNRP allowed assessment of intra-donor changes. Together, these features support the feasibility of using donor-derived miRNAs to capture dynamic aspects of graft biology.

In transplantable grafts, pre-procurement serum miRNA profiles were similar between DBD and DCD donors suggesting that, in carefully selected grafts, the systemic inflammatory and hemodynamic changes associated with brain death may not translate into major differences in circulating liver-specific miRNAs. This is notable given that brain death induces a systemic pro-inflammatory response involving cytokine release, complement activation, endothelial dysfunction, and leukocyte recruitment, and has been associated with higher circulating inflammatory mediators and immunogenic extracellular vesicles compared with DCD and living donation [6, 7, 27].

Differences in miRNA expression between donor types became more evident in preservation fluid, where DCD grafts recovered with aNRP showed lower expression of miR-148a, miR-222, and miR-22. Previous studies have identified a preservation-fluid injury signature characterized by increased miR-122 expression and higher miR-122/222 ratios. This signature has been reported in conventionally recovered DCD grafts (without aNRP) compared with DBD grafts, as well as in grafts that subsequently develop early allograft dysfunction or ischemic-type biliary lesions, irrespective of donor type [10, 12]. These findings support preservation fluid as a promising source of graft-derived biomarkers.

Importantly, all DCD grafts included in the present study underwent abdominal normothermic regional perfusion before organ retrieval and preservation-fluid collection. Unlike conventionally recovered DCD grafts, these organs are exposed to a period of oxygenated reperfusion and metabolic recovery before preservation, allowing restoration of oxygen delivery, mitochondrial function, and ATP stores, together with partial recovery from warm ischemic injury [28]. Consequently, preservation-fluid miRNA profiles obtained after aNRP should not be expected to mirror those reported in conventionally recovered DCD grafts without regional perfusion. Therefore, the present findings should not be interpreted as contradicting previous reports in conventionally recovered DCD grafts but rather as reflecting the distinct biological environment created by regional reperfusion before organ preservation.

Against this background, the lower expression of miR-148a, miR-222, and miR-22 observed in DCD grafts recovered with aNRP in the present study may initially appear counterintuitive if interpreted solely in the context of warm ischemic injury. However, unlike serum or liver tissue, preservation fluid represents a procedure-derived compartment. Consequently, miRNA concentrations in preservation fluid are likely to reflect the combined effects of intracellular miRNA abundance, active extracellular secretion, passive release from injured cells, reperfusion during aNRP and vascular washout during graft flushing, rather than the magnitude of hepatocellular injury alone. Preservation fluid miRNA profiles should therefore not be interpreted as direct surrogates of injury severity, but rather as molecular readouts influenced by both biological responses and procurement-related processes.

Notably, the direction of change observed for miR-148a and miR-222 was internally consistent across preservation fluid and DCD serum collected after warm ischemia during aNRP, suggesting that these findings are unlikely to reflect technical variation. Experimental and clinical studies have suggested reduced tissue miR-148a following prolonged warm ischemia [17] and decreased perfusate miR-222 expression in grafts developing ischemic-type biliary lesions [12], supporting the biological plausibility of these observations. Nevertheless, the precise mechanisms underlying these compartment-specific expression patterns remain uncertain. Future studies incorporating signal sampling during regional perfusion and machine perfusion will be required to distinguish changes related to intracellular regulation, selective extracellular release, and passive leakage following cellular injury.

By contrast, the exploratory analysis of the three declined steatotic grafts identified miRNA expression patterns that were biologically compatible with increased hepatocellular injury. Compared with transplantable DBD grafts, these grafts showed lower miR-122 expression in liver tissue together with higher circulating miR-122 and miR-122/miR-222 ratios, findings that align with the known vulnerability of steatotic livers to ischemia-reperfusion injury [14] and with previously reported miRNA expression patterns in hepatic injury [12, 29, 30]. Because donor serum samples were obtained before procurement, the increased circulating miR-122 observed in discarded steatotic grafts may reflect either greater susceptibility to the inflammatory and hemodynamic changes associated with brain death [5–7], resulting in enhanced hepatocellular injury before organ retrieval, or intrinsic alterations in miR-122 regulation associated with hepatic steatosis, as both reduced hepatic miR-122 expression and increased circulating miR-122 have previously been described in steatotic liver disease [29, 30]. The present study cannot distinguish between these complementary mechanisms. Nevertheless, the observation that these differences were already present in donor serum before graft procurement raises the possibility that circulating miRNAs may provide biologically relevant information regarding graft quality. If confirmed in larger cohorts, these observations may support further investigation of donor-derived circulating miRNAs as complementary molecular biomarkers for pre-procurement graft assessment. However, given the limited number of declined grafts and the absence of transplantation outcomes, these observations should be interpreted cautiously and considered hypothesis-generating. Future studies including larger cohorts of marginal donor livers will be required to validate these findings and define their potential role in assessment of graft quality.

The observed differences in miRNA expression across compartments are biologically plausible. miR-122, the most abundant liver-specific miRNA, is a well-established marker of hepatocellular injury [17, 31], whereas miR-222 has been linked to cholangiocyte and endothelial function [9, 32], and miR-155 to inflammatory and immune signaling pathways [11, 33, 34]. The inverse association between preservation-fluid miR-155 and early transaminase release is consistent with the recognized role of miR-155 in inflammatory and immune-related pathways. However, because this observation emerged from an exploratory analysis without adjustment for multiple testing, it should be interpreted cautiously and requires independent validation. Importantly, associations between donor-derived miRNAs and early post-transplant injury were limited, with only preservation-fluid miR-155 showing a statistically significant correlation with transaminase peaks. While other miRNAs showed directional trends, no consistent predictive pattern emerged.

Notably, all grafts in this study were preserved using the same solution (IGL-1), allowing for a standardized assessment of miRNA expression across samples and minimizing variability related to preservation-associated redox effects [35]. The use of IGL-1, which has been shown to attenuate ischemia–reperfusion injury, may partly explain the modest magnitude of these associations [36].

The present study should be interpreted within the context of its design. Although the overall donor cohort was limited, the exploratory analysis of declined steatotic grafts was based on only three cases and should therefore be interpreted with particular caution. Nevertheless, several methodological aspects strengthen the robustness of the observations. First, the use of standardized RT-qPCR techniques with validated normalization strategies resulted in low technical variability. Second, multi-compartment analysis increased the amount of information obtained per donor. Finally, the inclusion of partially paired samples, particularly in DCD donors before and during aNRP, allowed assessment of intra-donor changes, reducing the impact of interindividual variability.

At the same time, this study was designed as an exploratory analysis, and the findings should be interpreted accordingly. The targeted miRNA panel was based on prior literature and may not capture the full spectrum of relevant molecular signals. Sampling was performed at defined time points, which may not fully reflect the temporal dynamics of injury and recovery. Furthermore, mechanistic interpretation of the observed compartment-specific miRNA expression patterns was limited by the absence of correlations with established biochemical, inflammatory, and tissue injury biomarkers. Future studies should integrate these complementary biomarkers to improve biological interpretation and validate the clinical relevance of donor-derived miRNA profiling. In addition, the absence of an external validation cohort limits the generalizability of the findings.

In conclusion, this study demonstrates the feasibility of multi-compartment miRNA profiling in deceased liver donors and highlights the value of integrating tissue-, fluid-, and serum-derived molecular information to characterize graft biology. The compartment-specific patterns observed here provide a framework for future studies investigating molecular graft assessment.

## Data Availability

A summary of the data underlying the reported analyses, including assay availability and Ct values across the analysed compartments, is provided in the Supplementary Material. The complete raw dataset is available from the corresponding authors upon reasonable request.

## References

[1] Organización Nacional de Trasplantes. Actividad de donación y trasplante hepático en España 2024 (2025). Available online at: https://www.ont.es/wp-content/uploads/2025/03/ACTIVIDAD-DE-DONACION-Y-TRASPLANTE-HEPATICO-ESPANA-2024.pdf (Accessed April 14, 2025).

[2] Tang JX Na N Li JJ Fan L Weng RH Jiang N . Outcomes of controlled donation after cardiac death compared with donation after brain death in liver transplantation: a systematic review and meta-analysis. Transpl Proc (2018) 41:33–41. 10.1016/j.transproceed.2017.11.034 29407328

[3] Savier E Lim C Rayar M Orlando F Boudjema K Mohkam K Favorable outcomes of liver transplantation from controlled circulatory death donors using normothermic regional perfusion compared to brain death donors. Transplantation (2020) 104(9):1943–51. 10.1097/TP.0000000000003372 32639402

[4] Ruiz P Valdivieso A Palomares I Prieto M Ventoso A Salvador P Similar results in liver transplantation from controlled donation after circulatory death donors with normothermic regional perfusion and donation after brain death donors: a case-matched single-center study. Liver Transpl (2021) 27(12):1747–57. 10.1002/lt.26281 34455694

[5] Golling M Mehrabi A Blum K Jahnke C Kellner H Bud O Effects of hemodynamic instability on brain death-induced prepreservation liver damage. Transplantation (2003) 75(8):1154–9. 10.1097/01.TP.0000062868.34247.8F 12717195

[6] Weiss S Kotsch K Francuski M Reutzel-Selke A Mantouvalou L Klemz R Brain death activates donor organs and is associated with a worse I/R injury after liver transplantation. Am J Transpl (2007) 7(6):1584–93. 10.1111/j.1600-6143.2007.01799.x 17430397

[7] Sandiumenge A Bello I Coll-Torres E Gomez-Brey A Franco-Jarava C Miñambres E Systemic inflammation differences in brain-vs. Circulatory-dead donors: impact on lung transplant recipients. Transpl Int (2024) 37:12512. 10.3389/ti.2024.12512 38887494 PMC11182341

[8] Verhoeven CJ Farid WRR De Jonge J Metselaar HJ Kazemier G Van Der Laan LJW . Biomarkers to assess graft quality during conventional and machine preservation in liver transplantation. J Hepatol (2014) 61(3):672–84. 10.1016/j.jhep.2014.04.031 24798616

[9] Matton APM Selten JW Roest HP de Jonge J Ijzermans JNM de Meijer VE Cell-free microRNAs as early predictors of graft viability during *ex vivo* normothermic machine perfusion of human donor livers. Clin Transpl (2020) 34(3):e13790. 10.1111/ctr.13790 31984571 PMC7154637

[10] Selten JW Verhoeven CJ Heedfeld V Roest HP de Jonge J Pirenne J The release of microRNA-122 during liver preservation is associated with early allograft dysfunction and graft survival after transplantation. Liver Transpl (2017) 23(7):946–56. 10.1002/lt.24766 28388830

[11] Millán O Ruiz P Orts L Ferré P Crespo G Santana M Monitoring of miR-181a-5p and miR-155-5p plasmatic expression as prognostic biomarkers for acute and subclinical rejection in *de novo* adult liver transplant recipients. Front Immunol (2019) 10:873. 10.3389/fimmu.2019.00873 31068943 PMC6491707

[12] Verhoeven CJ Farid WRR De Ruiter PE Hansen BE Roest HP De Jonge J MicroRNA profiles in graft preservation solution are predictive of ischemic-type biliary lesions after liver transplantation. J Hepatol (2013) 59(6):1231–8. 10.1016/j.jhep.2013.07.034 23928409

[13] Dalmau M Charco R Bilbao I Dopazo C Caralt M Molino JA Heparinase I treatment to overcome RNA quantification interference in heparinized liver donor samples: one size fits all? PLoS One (2025) 20(5):e0322899. 10.1371/journal.pone.0322899 40354476 PMC12068581

[14] Hessheimer AJ Hartog H Marcon F Schlegel A Adam R Alwayn I Deceased donor liver utilisation and assessment: consensus guidelines from the European liver and intestine transplant association. J Hepatol (2025) 82(6):1089–109. 10.1016/j.jhep.2025.01.042 40189968

[15] Kleiner DE Brunt EM Van Natta M Behling C Contos MJ Cummings OW Design and validation of a histological scoring system for nonalcoholic fatty liver disease. Hepatology (2005) 41(6):1313–21. 10.1002/hep.20701 15915461

[16] Verhoeven CJ Selten JW Roest HP Farid WRR de Ruiter PE Hansen BE Corrigendum to “MicroRNA profiles in graft preservation solution are predictive of ischemic-type biliary lesions after liver transplantation” [J Hepatol 2013;59:1231–1238]. J Hepatol (2017) 67(6):1358–9. 10.1016/j.jhep.2017.09.001 23928409

[17] Farid WRR Pan Q Van Der Meer AJP De Ruiter PE Ramakrishnaiah V De Jonge J Hepatocyte-derived microRNAs as serum biomarkers of hepatic injury and rejection after liver transplantation. Liver Transpl (2012) 18(3):290–7. 10.1002/lt.22438 21932376

[18] Asaoka T Hernandez D Tryphonopoulos P Tekin A Garcia J Nishida S Clinical significance of intragraft miR-122 and -155 expression after liver transplantation. Hepatol Res (2014) 45:1–8. 10.1111/hepr.12424 25220676

[19] Schmuck RB Reutzel-Selke A Raschzok N Morgul HM Struecker B Lippert S Bile: miRNA pattern and protein-based biomarkers may predict acute cellular rejection after liver transplantation. Biomarkers (2017) 22(1):19–27. 10.1080/1354750X.2016.1201538 27344923

[20] Li Y Zhang L Liu F Xiang G Jiang D Pu X . Identification of endogenous controls for analyzing serum exosomal miRNA in patients with hepatitis B or hepatocellular carcinoma. Dis Markers (2015) 2015:893594. 10.1155/2015/893594 25814782 PMC4357047

[21] Shen J Wang Q Gurvich I Remotti H Santella RM . Evaluating normalization approaches for the better identification of aberrant microRNAs associated with hepatocellular carcinoma. Hepatoma Res (2016) 2(11):305–15. 10.20517/2394-5079.2016.28 28393113 PMC5380371

[22] Olthoff KM Kulik L Samstein B Kaminski M Abecassis M Emond J Validation of a current definition of early allograft dysfunction in liver transplant recipients and analysis of risk factors. Liver Transpl (2010) 16:943–9. 10.1002/lt.22091 20677285

[23] Galvin Z McDonough A Ryan J Stewart S . Blood alanine aminotransferase levels >1,000 IU/l - causes and outcomes. Clin Med (2015) 15(3):244–7. 10.7861/clinmedicine.15-3-244 26031973 PMC4953107

[24] Livak KJ Schmittgen TD . Analysis of relative gene expression data using real-time quantitative PCR and the 2−ΔΔCT method. Methods (2001) 25(4):402–8. 10.1006/meth.2001.1262 11846609

[25] Mestdagh P Van Vlierberghe P De Weer A Muth D Westermann F Speleman F A novel and universal method for microRNA RT-qPCR data normalization. Genome Biol (2009) 10(6):R64. 10.1186/gb-2009-10-6-r64 19531210 PMC2718498

[26] Andersen CL Jensen JL Falck Ørntoft T . Normalization of real-time quantitative reverse transcription-PCR data: a model-based variance estimation approach to identify genes suited for normalization, applied to bladder and colon cancer data sets. Cancer Res (2004) 64:5245–50. 10.1158/0008-5472.CAN-04-0496 15289330

[27] Ravichandran R Itabashi Y Zhou F Lin Y Mohanakumar T Chapman WC . Circulating exosomes from brain death and cardiac death donors have distinct molecular and immunologic properties: a pilot study. Clin Transpl (2023) 37(10):e15067. 10.1111/ctr.15067 37428019 PMC11019898

[28] Hessheimer AJ Riquelme F Fundora-Suárez Y García Pérez R Fondevila C . Normothermic perfusion and outcomes after liver transplantation. Transpl Rev (2019) 33(4):200–8. 10.1016/j.trre.2019.06.001 31239189

[29] Pirola CJ Gianotti TF Castaño GO Mallardi P Martino JS Ledesma MMGL Circulating microRNA signature in non-alcoholic fatty liver disease: from serum non-coding RNAs to liver histology and disease pathogenesis. Gut (2015) 64(5):800–12. 10.1136/gutjnl-2014-306996 24973316 PMC4277726

[30] Cheung O Puri P Eicken C Contos MJ Mirshahi F Maher JW Nonalcoholic steatohepatitis is associated with altered hepatic microRNA expression. Hepatology (2008) 48(6):1810–20. 10.1002/hep.22569 19030170 PMC2717729

[31] Roderburg C Benz F Vargas Cardenas D Koch A Janssen J Vucur M Elevated miR-122 serum levels are an independent marker of liver injury in inflammatory diseases. Liver Int (2015) 35(4):1172–84. 10.1111/liv.12627 25039534

[32] Pisarello MJL Loarca L Ivanics T Morton L LaRusso N . MicroRNAs in the cholangiopathies: pathogenesis, diagnosis, and treatment. J Clin Med (2015) 4(9):1688–712. 10.3390/jcm4091688 26343736 PMC4600153

[33] Rodriguez A Vigorito E Clare S Warren MV Couttet P Soond DR Requirement of bic/microRNA-155 for normal immune function. Science (2007) 316(5824):608–11. 10.1126/science.1139253 17463290 PMC2610435

[34] Hu J Huang S Liu X Zhang Y Wei S Hu X . MiR-155: an important role in inflammation response. J Immunol Res (2022) 2022:7437281. 10.1155/2022/7437281 35434143 PMC9007653

[35] Adam R Delvart V Karam V Ducerf C Navarro F Letoublon C Compared efficacy of preservation solutions in liver transplantation: a long-term graft outcome study from the European Liver Transplant Registry. Am J Transpl (2015) 15(2):395–406. 10.1111/ajt.13060 25612492

[36] Mosbah IB Zaouali M Martel C Bjaoui M Abdennebi HB Hotter G IGL-1 solution reduces endoplasmic reticulum stress and apoptosis in rat liver transplantation. Cell Death Dis (2012) 3(12):e340. 10.1038/cddis.2012 22402603 PMC3317344

