# Supplementary material for: Multi-compartment profiling of donor-derived microRNAs during liver graft procurement: a prospective feasibility study
Source: Transpl Int. 2026 Oct 6;39:17090. doi: 10.3389/ti.2026.17090 (PMC13682442; doi:10.3389/ti.2026.17090)
Supplement: Supplementary file 1 [file Supplementaryfile1.docx]

**Supplementary Material**

**Table S1. Ct values of miRNAs in donor liver biopsies from transplantable grafts.**

(A) Mean cycle threshold (Ct) values and standard deviations (SD) for selected endogenous miRNAs and the spike-in control cel-miR-39 across twenty liver donors. (B) Mean Ct values and standard deviations for endogenous miRNAs and cel-miR-39, stratified by donor type: DBD (n = 10) and DCD (n = 10).

**A.**

| **miRNAs** | **n** | **Ct mean** | **Ct SD** |
| --- | --- | --- | --- |
| miR-122 | 20 | 13.46 | 0.57 |
| miR-148a | 20 | 18.54 | 0.03 |
| miR-22 | 20 | 18.86 | 0.36 |
| miR-222 | 20 | 23.83 | 0.64 |
| miR-155 | 20 | 25.76 | 0.77 |
| miR-103a | 20 | 19.78 | 0.58 |
| miR-191 | 20 | 20.33 | 0.55 |
| miR-30c | 20 | 19.37 | 0.60 |
| miR-16 | 20 | 18.65 | 0.58 |
| miR-let7a | 20 | 16.95 | 0.62 |
| cel-miR-39 | 20 | 28.39 | 0.35 |

**B.**

| **Donor group** | **miRNAs** | **n** | **Ct mean** | **Ct SD** |
| --- | --- | --- | --- | --- |
| DBD | miR-122 | 10 | 13.63 | 0.53 |
|  | miR-148a | 10 | 18.72 | 0.61 |
|  | miR-22 | 10 | 18.96 | 0.39 |
|  | miR-222 | 10 | 24.08 | 0.56 |
|  | miR-155 | 10 | 26.08 | 0.66 |
|  | miR-103a | 10 | 20.04 | 0.44 |
|  | miR-191 | 10 | 20.45 | 0.49 |
|  | miR-30c | 10 | 19.55 | 0.55 |
|  | miR-16 | 10 | 18.46 | 0.64 |
|  | miR-let7a | 10 | 17.31 | 0.46 |
|  | cel-miR-39 | 10 | 28.52 | 0.17 |
| DCD | miR-122 | 10 | 13.29 | 0.59 |
|  | miR-148a | 10 | 18.36 | 0.67 |
|  | miR-22 | 10 | 18.76 | 0.32 |
|  | miR-222 | 10 | 23.59 | 0.65 |
|  | miR-155 | 10 | 25.44 | 0.76 |
|  | miR-103a | 10 | 19.52 | 0.61 |
|  | miR-191 | 10 | 20.21 | 0.61 |
|  | miR-30c | 10 | 19.19 | 0.63 |
|  | miR-16 | 10 | 18.46 | 0.64 |
|  | miR-let7a | 10 | 16.59 | 0.56 |
|  | cel-miR-39 | 10 | 28.26 | 0.43 |

**Table S2. Ct values of miRNAs in donor preservation fluid from transplantable grafts.**

(A) Mean Ct values and standard deviations for selected endogenous miRNAs and the spike-in control cel-miR-39 across twenty liver donors. (B) Mean Ct values and standard deviations for endogenous miRNAs and cel-miR-39, stratified by donor type: DBD (n = 10) and DCD (n = 10)

**A.**

| **miRNAs** | **n** | **Ct mean** | **Ct SD** |
| --- | --- | --- | --- |
| miR-122 | 20 | 27.45 | 2.13 |
| miR-148a | 20 | 32.26 | 2.07 |
| miR-22 | 20 | 29.60 | 2.25 |
| miR-222 | 20 | 32.39 | 2.30 |
| miR-155 | 13 | 35.01 | 2.25 |
| miR-103a | 20 | 32.13 | 3.15 |
| miR-191 | 20 | 31.69 | 2.91 |
| miR-30c | 20 | 32.57 | 2.68 |
| miR-16 | 20 | 28.48 | 3.63 |
| miR-let7a | 20 | 29.39 | 3.06 |
| cel-miR-39 | 20 | 28.23 | 0.37 |
| UniSp4 | 20 | 30.10 | 1.46 |

**B.**

| **Donor group** | **miRNAs** | **n** | **Ct mean** | **Ct SD** |
| --- | --- | --- | --- | --- |
| DBD | miR-122 | 10 | 27.82 | 1.83 |
|  | miR-148a | 10 | 32.56 | 1.68 |
|  | miR-22 | 10 | 30.13 | 1.73 |
|  | miR-222 | 10 | 32.99 | 1.23 |
|  | miR-155 | 6 | 36.31 | 1.06 |
|  | miR-103a | 10 | 33.40 | 2.11 |
|  | miR-191 | 10 | 32.65 | 1.95 |
|  | miR-30c | 10 | 33.19 | 2.19 |
|  | miR-16 | 10 | 30.12 | 1.68 |
|  | miR-let7a | 10 | 30.41 | 2.33 |
|  | cel-miR-39 | 10 | 28.15 | 0.43 |
|  | UniSp4 | 10 | 29.87 | 1.51 |
| DCD | miR-122 | 10 | 27.08 | 2.43 |
|  | miR-148a | 10 | 31.95 | 2.46 |
|  | miR-22 | 10 | 29.08 | 2.66 |
|  | miR-222 | 10 | 31.77 | 2.96 |
|  | miR-155 | 7 | 33.90 | 2.46 |
|  | miR-103a | 10 | 30.85 | 3.59 |
|  | miR-191 | 10 | 30.72 | 3.47 |
|  | miR-30c | 10 | 31.95 | 3.08 |
|  | miR-16 | 10 | 26.85 | 4.37 |
|  | miR-let7a | 10 | 28.37 | 3.48 |
|  | cel-miR-39 | 10 | 28.30 | 0.31 |
|  | UniSp4 | 10 | 30.32 | 1.46 |

**Table S3.** **Ct values of miRNAs in donor serum** **from transplantable grafts.**

(A) Mean Ct values and standard deviations for selected endogenous miRNAs and the spike-in control cel-miR-39 across twenty liver donors. (B) Mean Ct values and standard deviations for endogenous miRNAs and cel-miR-39, stratified by donor type: DBD (n = 10) and paired DCD samples at T0 and T1 (n = 10).

**A.**

| **miRNAs** | **n** | **Ct mean** | **Ct SD** |
| --- | --- | --- | --- |
| miR-122 | 30 | 30.94 | 2.04 |
| miR-148a | 30 | 31.47 | 2.46 |
| miR-22 | 30 | 27.54 | 2.27 |
| miR-222 | 27 | 30.56 | 2.58 |
| miR-155 | 26 | 35.39 | 1.68 |
| miR-103a | 26 | 30.79 | 2.26 |
| miR-191 | 30 | 28.86 | 1.98 |
| miR-30c | 29 | 31.43 | 2.21 |
| miR-16 | 30 | 23.97 | 2.16 |
| miR-let7a | 30 | 28.58 | 2.32 |
| cel-miR-39 | 30 | 30.02 | 0.33 |
| UniSp4 | 30 | 26.78 | 2.52 |

**B.**

| **Donor group** | **miRNAs** | **n** | **Ct mean** | **Ct SD** |
| --- | --- | --- | --- | --- |
| DBD | miR-122 | 10 | 31.22 | 2.30 |
|  | miR-148a | 10 | 30.55 | 1.67 |
|  | miR-22 | 10 | 26.83 | 1.28 |
|  | miR-222 | 10 | 29.67 | 1.42 |
|  | miR-155 | 9 | 35.65 | 1.72 |
|  | miR-103a | 10 | 30.17 | 1.81 |
|  | miR-191 | 10 | 28.97 | 1.57 |
|  | miR-30c | 10 | 30.89 | 1.49 |
|  | miR-16 | 10 | 23.67 | 1.70 |
|  | miR-let7a | 10 | 28.03 | 1.68 |
|  | cel-miR-39 | 10 | 29.89 | 0.13 |
|  | UniSp4 | 10 | 25.37 | 1.24 |
| DCD-T0 | miR-122 | 10 | 30.41 | 2.01 |
|  | miR-148a | 10 | 30.77 | 1.93 |
|  | miR-22 | 10 | 26.75 | 1.74 |
|  | miR-222 | 10 | 30.36 | 2.85 |
|  | miR-155 | 10 | 35.37 | 1.56 |
|  | miR-103a | 10 | 30.72 | 2.39 |
|  | miR-191 | 10 | 28.06 | 1.71 |
|  | miR-30c | 10 | 30.82 | 1.67 |
|  | miR-16 | 10 | 23.49 | 2.07 |
|  | miR-let7a | 10 | 27.77 | 1.41 |
|  | cel-miR-39 | 10 | 29.98 | 0.28 |
|  | UniSp4 | 10 | 26.00 | 1.68 |
| DCD-T1 | miR-122 | 10 | 31.20 | 1.88 |
|  | miR-148a | 10 | 33.10 | 2.93 |
|  | miR-22 | 10 | 29.06 | 2.85 |
|  | miR-222 | 7 | 32.11 | 3.09 |
|  | miR-155 | 7 | 35.07 | 1.97 |
|  | miR-103a | 6 | 31.96 | 2.62 |
|  | miR-191 | 10 | 29.54 | 2.45 |
|  | miR-30c | 9 | 32.70 | 2.97 |
|  | miR-16 | 10 | 24.76 | 2.60 |
|  | miR-let7a | 10 | 29.94 | 3.06 |
|  | cel-miR-39 | 10 | 30.18 | 0.45 |
|  | UniSp4 | 10 | 28.97 | 2.82 |

**Table S4.** **Ct values of miRNAs from steatotic grafts declined for transplantation (exploratory analysis).**

Mean Ct values and standard deviations for endogenous miRNAs and the spiked-in control cel-miR-39 Ct in three donors with moderate-severe steatotic grafts, stratified by sample type.

| **Sample** | **miRNAs** | **n** | **Ct mean** | **Ct SD** |
| --- | --- | --- | --- | --- |
| Liver tissue | miR-122 | 3 | 21.18 | 0.55 |
|  | miR-148a | 3 | 25.42 | 0.54 |
|  | miR-22 | 3 | 25.51 | 0.31 |
|  | miR-222 | 3 | 30.61 | 0.35 |
|  | miR-155 | 3 | 32.70 | 0.88 |
|  | miR-103a | 3 | 25.72 | 0.42 |
|  | miR-191 | 3 | 27.38 | 0.42 |
|  | miR-30c | 3 | 26.11 | 0.65 |
|  | miR-16 | 3 | 25.16 | 0.65 |
|  | miR-let7a | 3 | 24.32 | 0.61 |
|  | cel-miR-39 | 3 | 29.13 | 0.03 |
| Preservation fluid | miR-122 | 3 | 26.98 | 4.63 |
|  | miR-148a | 3 | 30.68 | 4.57 |
|  | miR-22 | 3 | 29.49 | 5.22 |
|  | miR-222 | 3 | 33.73 | 4.44 |
|  | miR-155 | 3 | 31.71 | 5.44 |
|  | miR-103a | 3 | 30.94 | 4.60 |
|  | miR-191 | 3 | 32.69 | 4.81 |
|  | miR-30c | 3 | 32.03 | 4.63 |
|  | miR-16 | 3 | 29.92 | 4.82 |
|  | miR-let7a | 3 | 30.46 | 5.61 |
|  | cel-miR-39 | 3 | 27.79 | 0.01 |
|  | UniSp4 | 3 | 30.87 | 2.78 |
| Serum | miR-122 | 3 | 27.09 | 2.30 |
|  | miR-148a | 3 | 28.62 | 2.67 |
|  | miR-22 | 3 | 26.41 | 2.27 |
|  | miR-222 | 3 | 30.17 | 1.19 |
|  | miR-155 | 3 | 35.33 | 0.36 |
|  | miR-103a | 3 | 27.91 | 0.93 |
|  | miR-191 | 3 | 28.94 | 1.01 |
|  | miR-30c | 3 | 30.57 | 0.93 |
|  | miR-16 | 3 | 23.97 | 1.05 |
|  | miR-let7a | 3 | 28.12 | 0.73 |
|  | cel-miR-39 | 3 | 27.74 | 0.03 |
|  | UniSp4 | 3 | 25.71 | 0.99 |

**Figure S1. Relationship between donor miRNA profiles and early allograft dysfunction (EAD).**

-ΔCt expression of miR-122, miR-148a, miR-222, miR-22 and miR-155 in n = 20 donors (DBD and DCD cohorts). miRNA expression is shown for liver tissue (A, D), preservation fluid (B, E), and serum samples collected prior to laparotomy (C, F).

**Figure S2.** **Selection of optimal reference genes for RT-qPCR normalization.**

(A) Pairwise variation (Vn/Vn+1) analysis used to determine the optimal number of reference genes required for normalization across different sample types. Expression stability rankings and plots are shown for each group using the geNorm method (B, C) and the NormFinder method (D, E).

**Figure S3. Normalization using endogenous and synthetic miRNA reference genes.**

Mean miRNA coefficient of variation (CV) values for each donor group and the cumulative distribution of CVs are shown for normalization using one (orange), two (green) and five (blue) endogenous miRNA reference genes, or the synthetic spike-in control cel-miR-39 (purple). Panels represent: (A) liver tissue; (B) preservation fluid; and (C) serum samples.
